# Simultaneous surgical management of renal cancer with atrial thrombotic extension and severe chronic coronary artery disease: a case report

**DOI:** 10.1186/s13256-023-04292-3

**Published:** 2023-12-13

**Authors:** Giovanni Battista Filomena, Filippo Marino, Eros Scarciglia, Pierluigi Russo, Fabrizio Fantasia, Riccardo Bientinesi, Mauro Ragonese, Nazario Foschi, Gaetano Gulino, Emilio Sacco, Marco Racioppi

**Affiliations:** Department of Urology, Fondazione Policlinico Universitario A. Gemelli IRCCS, Università Cattolica del Sacro Cuore, Rome, Italy

**Keywords:** Kidney, Renal cell carcinoma, Tumor thrombus, Nephrectomy, Cardiac thrombus, Chronic cardiac ischemia

## Abstract

**Background:**

Renal cell carcinoma accounts for 2–3% of all malignant cancers in adults and is characterized by the potential development of venous tumor thrombus.

**Case presentation:**

We present a rare case of a 62-year-old Caucasian man who arrived in the emergency department for monosymptomatic hematuria. Further investigation revealed a right renal cell carcinoma with 16 cm intravascular extension through the renal vein into the inferior vena cava and right atrium associated with significant coronary artery disease based on the computed tomography scan and coronary angiography. To the best of our knowledge, after an extensive literature review, only one similar case has been reported with involvement of the contralateral kidney. Therefore, there are no applicable management recommendations. After performing coronary artery bypass graft surgery, we proceeded with an open right radical nephrectomy and inferior vena cava and right atrium thrombectomy under cardiopulmonary bypass and while the patient’s heart was still beating. The postoperative course went without complications, and the patient was discharged from the hospital on the 10th postoperative day.

**Conclusions:**

Radical nephrectomy and thrombectomy with reconstruction of the inferior vena cava combined with coronary artery bypass graft can be performed safely and effectively in selected patients with renal cell carcinoma and significant coronary artery disease. Multidisciplinary teamwork and careful patient selection are essential for optimal outcomes.

## Background

Renal cell carcinoma (RCC) is an insidious neoplasm that accounts for around 2% of all cancer diagnoses and deaths globally and is expected to become more prevalent [1]. In Western countries, most cases of RCC are incidentally detected during imaging, typically with magnetic resonance imaging (MRI), ultrasound, or computed tomography (CT) scans. The “classic triad” of symptoms: hematuria, flank pain, and palpable masses occurs in just 10% of patients [2]. RCC naturally tends to grow into areas with the lowest level of resistance, as well as to directly infiltrate nearby structures, metastasize through the local lymph nodes, or disseminate hematogenously. It has been estimated that 4–10% of individuals undergoing nephrectomy to treat cancer have a direct extension of RCC into the inferior vena cava (IVC). In 0.3–1% of patients with RCC, tumor thrombi can extend into the right atrium [3, 4]. We present a rare case of a 62-year-old man with a kidney tumor and an extended intravascular development into the IVC up to his right atrium, which was associated with a history of advanced coronary heart disease.

## Case presentation

**Fig. 1:**
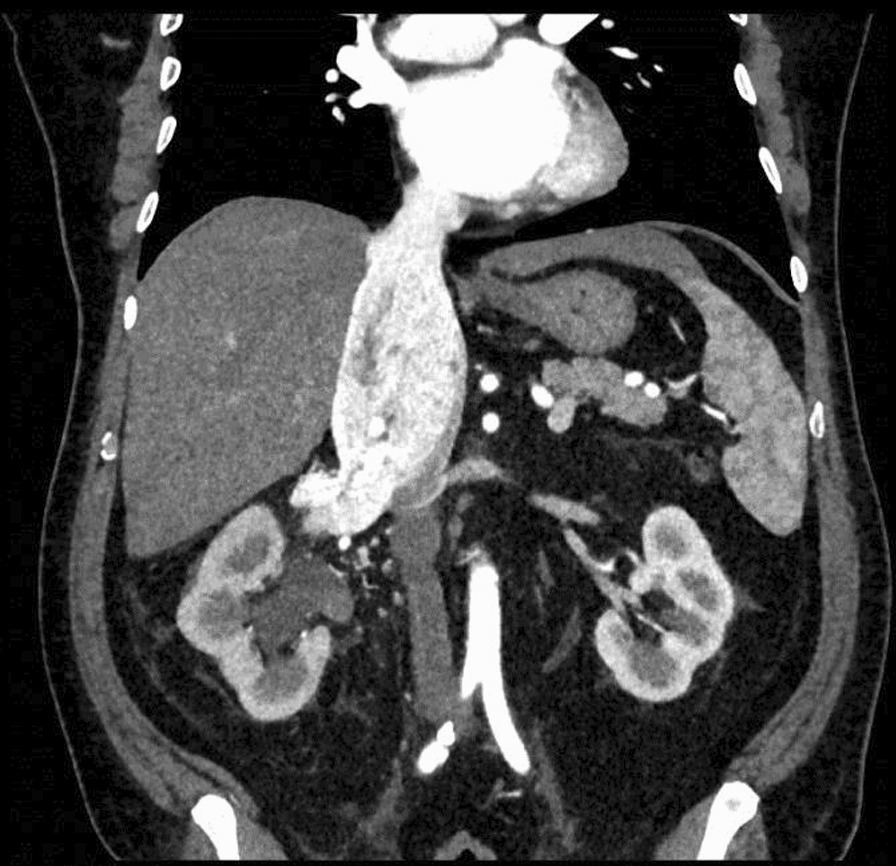
Preoperative computed tomography scan showing tumor mass and thrombus extension along inferior vena cava (coronal plane)

**Fig. 2:**
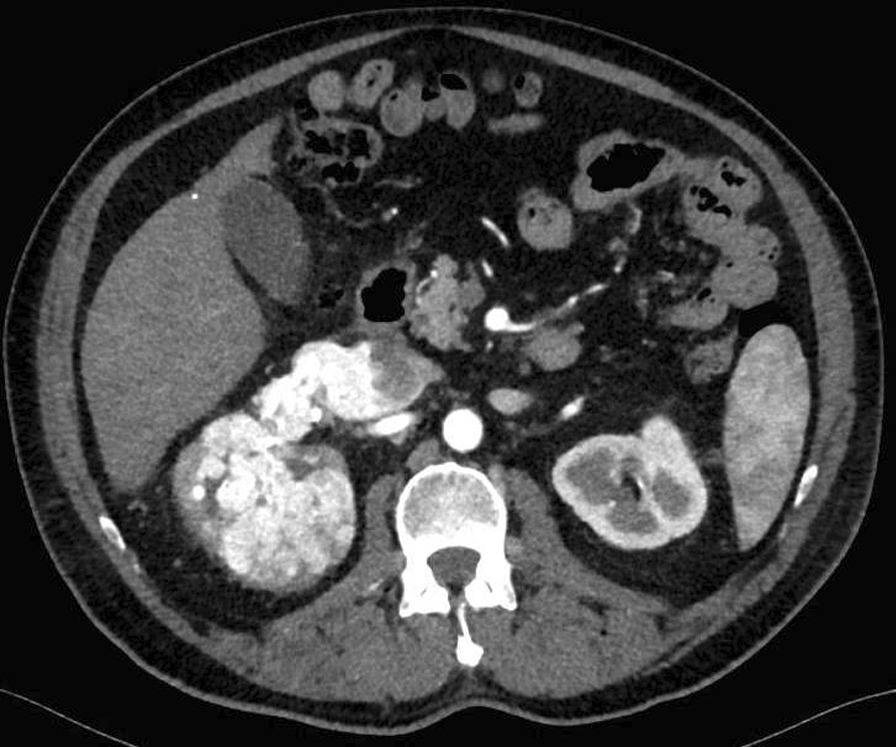
Preoperative computed tomography scan showing tumor mass and thrombus extension along inferior vena cava (axial plane)

A 62-year-old Caucasian male patient, with a height of 170 cm and a weight of 82 kg, presented with hematuria and was admitted to the emergency department of our institution. Abdominal ultrasound examination revealed a voluminous right renal mass that extended through the IVC. He denied having any pain, fever, or lower urinary tract symptoms (LUTS). On physical examination, the abdomen was globose with fat, no mass was palpable and the patient reported no previous abdominal surgery. CT scan confirmed the presence of a right renal tumor with intravascular extension into the IVC and right atrium. The tumor was classified as a type IV thrombus according to the Mayo classification [5]. MRI was consistent with the CT scan findings and no lymph node involvement, expansion to nearby organs, or distant metastases were detected on preoperative imaging, and clinical staging was T3cN0M0 (Figs. 1 and 2). The tumor thrombus in the right atrium was approximately 3.5 cm in size and extended from the right renal vein for about 16 cm.

**Fig. 3:**
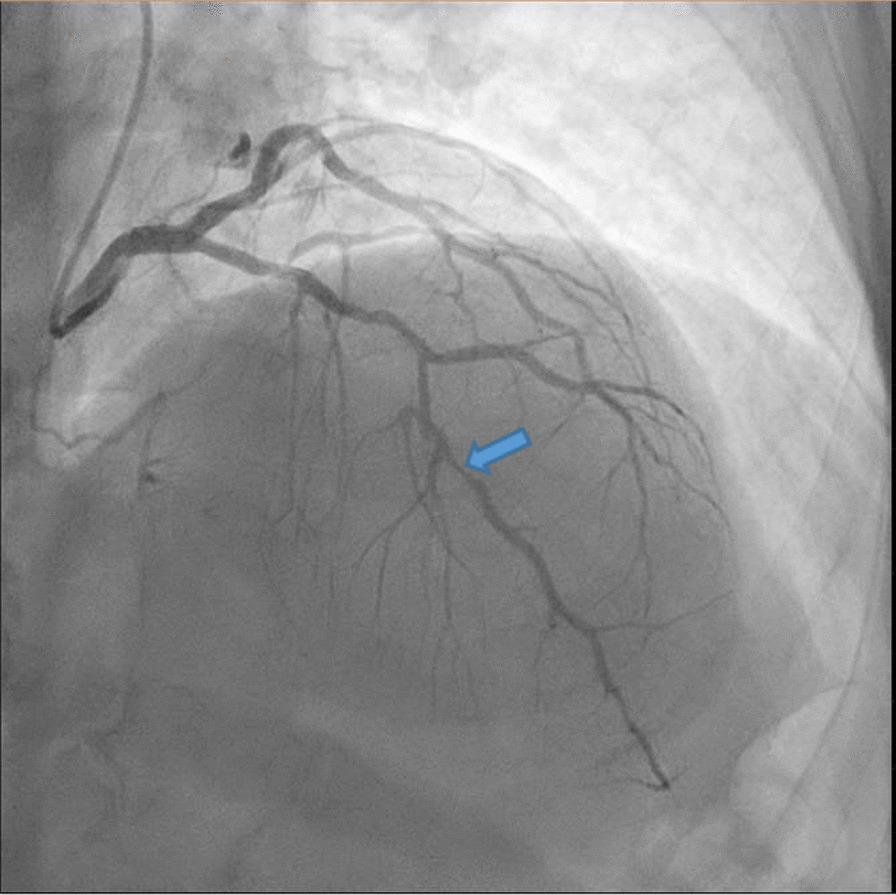
Preoperative coronary angiography showing stenosis of anterior descending coronary artery (The arrow indicates the location of the stenosis)

The patient had a medical history of type II diabetes mellitus on insulin therapy, dyslipidemia under medication, and hypertensive heart disease with an inferior myocardial infarction episode approximately 20 years ago. Coronary angiography (Fig. 3) indicated a localized 90% stenosis in the anterior descending coronary artery. Preoperative arterial and venous Doppler ultrasound of the carotid, jugular, and vertebral arteries and veins, as well as the upper and lower limbs, excluded thrombosis and assessed major vessel patency. Given the patient’s cardiac condition and the potential for intraoperative bleeding, urologic surgery carried a particularly high risk. However, due to the complex preoperative situation and the high risk of disease progression, postponing surgical intervention could be critical. Following an interdisciplinary discussion involving urologists and cardiac surgeons, a combined procedure was deemed appropriate.

A multidisciplinary team, comprising a cardiothoracic surgeon, a general surgeon (specialized in hepatobiliary surgery), and an urologist performed the surgical intervention. The patient was placed in lithotomy position and underwent median sternotomy, pericardial opening, and coronary artery bypass grafting (CABG) using the left internal mammary artery (LIMA) on the beating heart. The anterior wall of the chest was exposed with a tissue stabilizer, and an epicardial electrode was placed on the right atrium. After a subcostal incision Chevron type enlarged to the right, the inferior vena cava and right renal vein were isolated and fully exposed, the right gonadal vein, ureter, and right renal artery were identified, ligated, and cut. The surgical procedure involved the meticulous mobilization of the kidney, including the adrenal gland, by ligating the ureter and artery, leaving only the renal vein as the sole remaining attachment. The right renal vein and the inferior vena cava were markedly enlarged, with ecstatic collateral circles and intercavo-aortic lymphadenopathy due to the known venous thrombosis. Extracorporeal circulation was then established through cannulation of the superior vena cava and right common femoral vein for venous drainage, and the ascending aorta for arterial return.

**Fig. 4:**
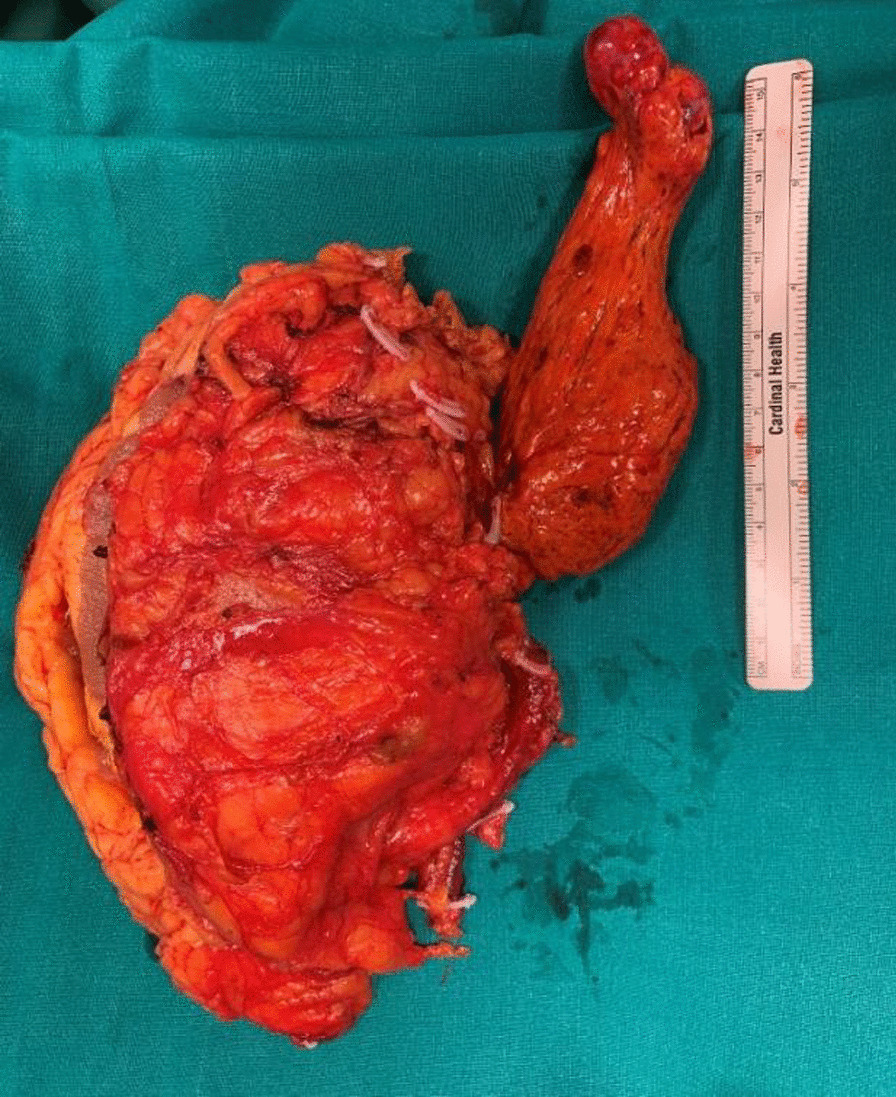
Right kidney with neoplasm and thrombus; surgical specimen

**Fig. 5:**
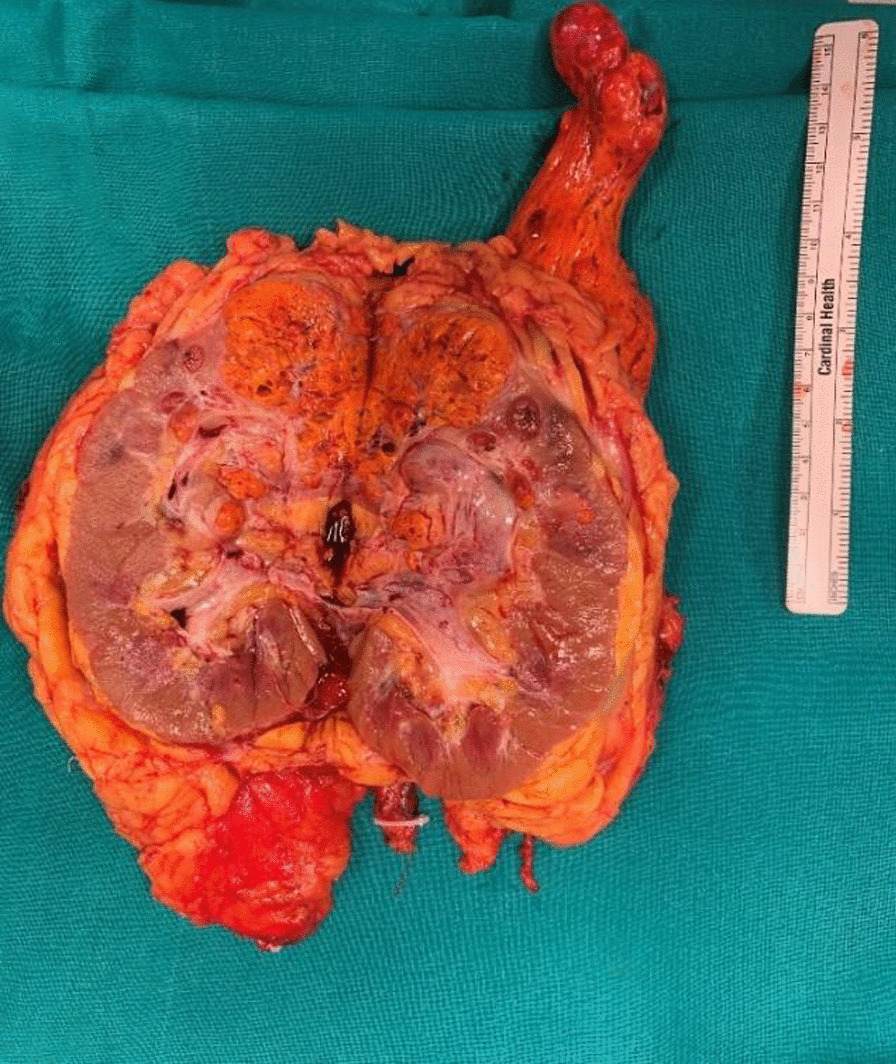
Dissected right kidney and neoplasm with thrombus; Surgical specimen

Following medial derotation of the liver, the caudal IVC, left renal vein, and hepatic peduncle were all completely clamped with vascular tourniquet to arrest any venous backflow. The renal vein was incised at the confluence with the vena cava; the cavotomy was extended cranially until the caval thrombus was completely extracted under transesophageal ultrasound control. Then, radical nephrectomy was completed, and the IVC was then reconstructed. Total surgery time was 600 minutes and the blood loss was approximately 2000 mL. During the operating time, 2 units of concentrated red blood cells were transfused. Pathological examination revealed renal cell carcinoma, clear cell subtype (Fuhrman grade 3) in the absence of tumor necrosis and sarcomatoid differentiation (Figs. 4 and 5).

From the first postoperative day, the patient received acetylsalicylic acid 100 mg therapy, which was deemed necessary due to the aortocoronary bypass that was performed. There were no significant incidents of postoperative bleeding, and prior to discharge, the patient underwent a series of diagnostic tests, including a transesophageal echocardiogram, chest X-ray, and electrocardiogram, which revealed no abnormalities or significant findings. On the 10th postoperative day, the patient was discharged from the hospital after an uncomplicated postoperative course in the ward.

The patient was then placed under close follow-up with a total body CT scan every 3 months; no other pharmacological therapies were necessary. At the 9-month follow-up visit, the patient was in good general clinical condition. Good recovery was observed after surgery, with an Eastern Cooperative Oncology Group (ECOG) performance status of 0; the patient was self-sufficient in his daily activities. The patient’s height was 170 cm, while his weight at the visit was recorded as 76 kg with a body mass index (BMI) of 26.30 kg/m^2^. We report only a slight anemia with hemoglobin equal to 9.9 mg/dL. On the control total body CT scan, there were no evident signs of disease recurrence.

## Discussion

Renal cell carcinoma can cause a specific complication in which the tumor grows into the IVC up to the right atrium. Accurately determining how far the tumor has grown is important because it affects the surgical approach. MRI is superior to CT scan in demonstrating the involvement of the caval wall and hepatic veins, which is critical when the tumor infiltrates the wall, and reconstruction of the vena cava may be necessary [6]. The diameter of the IVC and renal vein on CT scan or MRI can indicate whether the vessel wall has been invaded. While the prognosis is often poor for patients with metastasis, those who have an IVC thrombus without distant metastasis have a more favorable outlook [7]. The median life expectancy of a patient with untreated renal cell carcinoma and tumor thrombus is poor, estimated to be around 5 months. If there are other factors present such as metastases, or the disease is in a more advanced stage (pT3b or pT3c), the life expectancy may be even lower. There is disagreement in the published literature regarding the relationship between thrombus level and survival in patients who underwent surgery [8]. Patients who do not have distant metastasis at the time of presentation and undergo successful resection have a favorable prognosis. Prior studies have reported 5-year survival rates ranging from 32–64% for this particular patient cohort following surgical intervention. [9]

The effectiveness of presurgery therapy for patients with RCC and IVC is not well supported by evidence [10]. A prospective trial investigating the effectiveness of sorafenib or sunitinib after surgery for non-metastatic RCC with IVC tumor thrombus did not find any improvement in overall survival, but found improved disease free survival [11]. According to the 2021 guidelines by the European Association of Urology (EAU) for RCC, pembrolizumab, an immune checkpoint inhibitor that targets PD-1, is recommended with low certainty as an adjuvant therapy for patients who have undergone radical nephrectomy and have an intermediate to high risk of cancer recurrence. The use of pembrolizumab has been shown to improve disease-free survival (DFS) in these patients, as compared with using a placebo [hazard ratio (HR) 0.63, 95% confidence interval (CI) 0.50–0.80] [12, 13]. Zhang and colleagues conducted a comparative analysis between traditional radical nephrectomy and IVC thrombectomy with a liver mobilization technique and temporary IVC filter placement. The study findings demonstrated a significantly higher overall survival rate in patients who underwent the liver mobilization technique. [14]

Previous investigations have explored the potential benefits of preoperative renal artery embolization (PRAE) in patients diagnosed with stage T3 RCC. However, when compared with non-PRAE cohorts, PRAE was significantly associated with increased operating time, blood loss, and hospitalization duration, as well as a higher perioperative mortality rate (8.4% versus 3.4% for PRAE versus no PRAE, respectively, *p* value not stated). Nonetheless, PRAE did demonstrate a non-significant trend toward a reduced risk of mortality from any cause. [15]

Several studies have suggested that laparoscopic and robot-assisted laparoscopic IVC thrombectomy is a safe and viable option for the management of RCC with level II–III IVC thrombus, particularly in the hands of experienced surgeons [16]. However, initial reports of robotic nephrectomy with level IV caval thrombus were only recently published in 2019 and 2020 [17]. These procedures carry a high risk of complications such as vascular injury, hemorrhage, and thrombus shedding. Although robotic caval thrombectomy for levels III and IV thrombi has been shown to be feasible, it has not been widely adopted in clinical practice due to several factors including uncertain oncologic outcomes, rarity of cases and the time-consuming process of intraoperative patient repositioning with kidney tumor side up and robot redocking. [18]

Cardiopulmonary bypass (CPB) is used to treat level IV tumor thrombus and has proven to be extremely effective in reducing the risk of potentially fatal complications like bleeding or embolism. However, the use of CPB can cause platelet dysfunction and coagulopathy, leading to significant bleeding when combined with the raw, exposed retroperitoneal surface following a nephrectomy. Despite these challenges, CPB remains a standard procedure for treating patients with an adherent intraatrial thrombus, and many medical institutions utilize it for level III thrombi and beyond [9]. Due to its complexity, surgical interventions for managing tumor thrombus require an experienced team, consisting of anesthesiologists, cardiac surgeons, and urological surgeons with proficiency in different surgical techniques. For instance, in levels III and IV IVC thrombus, a Chevron type incision may be performed in combination with a sternotomy. This type of incision offers several benefits, including enabling access to the IVC, renal pedicle, and contralateral kidney through a single incision. However, warm ischemia of the left renal artery, used to prevent venous return, is an additional factor to consider that can increase the complexity of the surgical intervention.

A common concern is how to manage patients who have both coronary artery disease and oncological diseases. If cardiac surgery is performed before tumor resection, delaying the latter procedure may increase immunosuppression and potentially accelerate tumor growth and metastasis, in addition to increasing costs and requiring a second operation and anesthesia. Avoiding a second surgery and anesthesia has further benefits in terms of costs and risks. To address these concerns, we decided to perform a single surgery to manage both conditions. However, the long-term therapeutic benefits and survival rates of this approach require further investigation with larger sample sizes and longer follow-up periods.

Previously, Wang and colleagues reported a case with similarities to our own, in which a left-sided RCC was found to be associated with severe coronary artery stenosis. The patient underwent a combined surgical intervention involving coronary bypass [19]. However, to the best of our knowledge, this is the first reported case of concurrent right radical nephrectomy and right atrial thrombectomy performed on a beating heart while on cardiopulmonary bypass in a patient with both renal thrombus in the atrium and severe coronary artery stenosis. Despite these challenging conditions, our experienced surgical team successfully executed the procedure. Although this procedure carried a significant level of risk, a multidisciplinary approach was employed, which proved to be critical for safe and efficient handling of this complex surgical case. We are hopeful that our successful management of this case can serve as a model for managing similar urological and cardiac surgical procedures in the future.

## Conclusion

This case report highlights the feasibility and efficacy of a one-stage surgical treatment for right RCC with level IV intravenous tumor thrombus (IVTT) and concomitant severe coronary artery stenosis. An open treatment approach was chosen given the severity of the clinical case, the large mass that needed removal, and the need to reduce operating time. Due to cardiac involvement and the diseas’s progression, which necessitated prompt intervention, no neoadjuvant immunotherapy drugs were administered. Furthermore, the utilization of PRAE was deemed unsuitable due to the unfavorable outcomes reported in the literature for these kinds of large tumor masses. Personalized treatment strategies based on individual patient factors such as tumor biology, localization, and patient comorbidity were crucial to achieving successful outcomes. We are hopeful that this innovative surgical technique will enable a more patient-centered approach, thereby reducing potentially lethal peri- and postoperative complications.

## Data Availability

The Editor-in-Chief of this publication is granted access to all copies of the materials.

## Abbreviations

RCC: Renal cell carcinoma
VTT: Venous tumor thrombus
IVC: Inferior vena cava
CABG: Coronary artery bypass graft surgery
MRI: Magnetic resonance imaging
CT: Computed tomography
LUTS: Lower urinary tract symptoms
IVTT: Intravenous tumor thrombus
LIMA: Left internal mammary artery
EAU: European Association of Urology
DFS: Disease-free survival
PRAE: Preoperative renal artery embolization
ECOG: Eastern Cooperative Oncology Group
CPB: Cardiopulmonary bypass
